# Rapid and Accurate Diagnosis of Breast Cancer by Fine‐Needle Aspiration Biopsy Using the “Click‐to‐Sense” Method

**DOI:** 10.1002/cam4.71525

**Published:** 2026-02-13

**Authors:** Yuri Kitahara, Tomonori Tanei, Takaaki Hatano, Ambara R. Pradipta, Koji Morimoto, Tadasuke Nagatomo, Kaori Abe, Nanae Masunaga, Chieko Mishima, Tetsuhiro Yoshinami, Masami Tsukabe, Yoshiaki Sota, Tomohiro Miyake, Masafumi Shimoda, Yuichi Motoyama, Eiichi Morii, Katsunori Tanaka, Kenzo Shimazu

**Affiliations:** ^1^ Department of Breast and Endocrine Surgery, Graduate School of Medicine Osaka University Suita Japan; ^2^ Department of Chemical Science and Engineering, School of Materials and Chemical Technology Tokyo Institute of Technology Meguro Japan; ^3^ Department of Health Science, Faculty of Human Science Osaka International University Moriguchi Japan; ^4^ Biofunctional Synthetic Chemistry Laboratory RIKEN Cluster for Pioneering Research Wako Japan; ^5^ Department of Pathology Osaka University Suita Japan; ^6^ Department of Pathology (C3), Graduate School of Medicine Osaka University Suita Japan

**Keywords:** acrolein, biopsy, click‐to‐sense, fine‐needle, mammary neoplasms

## Abstract

**Background:**

We have previously demonstrated the value of the “click‐to‐sense” (CTS) assay, in which a fluorescent probe targeting acrolein can detect cancer cells and differentiate between malignant and benign lesions in breast tissue. In this study, we assessed the usefulness of the CTS assay for diagnosis of breast tumors by fine‐needle aspiration biopsy (FNAB).

**Methods:**

A total of 126 FNABs were performed on live tissue samples obtained by surgery (63 breast cancers, 31 benign breast tumors, and 32 normal breast glands). CTS reagents (CTS probe and Hoechst dye mixed with encapsulating agents) were added to the aspirated cells and placed on slides, which were then cover‐slipped and imaged under a fluorescence microscope. Another FNAB slide was prepared for each of the same live tissue samples, fixed in ethanol, and subjected to Papanicolaou (PAP) staining. The diagnostic accuracy of the CTS assay was compared with that of PAP staining by histopathological examination of permanent sections.

**Results:**

The CTS assay had a sensitivity of 92.1%, a specificity of 96.8%, and an accuracy of 94.4% (119/126 samples); the respective values for PAP staining were 98.4%, 89.8%, and 94.2% (114/121 samples). The insufficiency/inadequacy rate was 0% for the CTS assay and 4% for PAP staining (5/126 samples).

**Conclusion:**

The CTS assay is as accurate as PAP staining for FNAB of breast lesions. This assay has the potential to establish a new diagnostic method in cytopathology in the future, because it has a lower inadequacy rate and is simpler and less labor‐intensive and time‐consuming to perform.

## 1 Introduction

Breast cancer is the leading malignancy in women in terms of both incidence and mortality [1], and early detection and accurate diagnosis of breast lesions are important in terms of subsequent therapeutic strategies [2]. The two most common techniques used for pathological diagnosis of breast lesions are core needle biopsy (CNB) for histological examination and fine‐needle aspiration biopsy (FNAB) for cytopathological examination, both of which have specific advantages and limitations [3]. Histological diagnosis by CNB provides detailed information over and above cell morphology, including tissue structure and intercellular relationships, thereby offering higher diagnostic accuracy in comparison with FNAB [3]. However, CNB is an invasive procedure that must be performed under local anesthesia and imposes a significant burden on patients in terms of discomfort, a long processing time, and high cost [4].

In contrast, FNAB with Papanicolaou (PAP) staining is a simpler, less expensive procedure with a lower risk of complications and a high patient acceptance rate owing to its non‐invasiveness and causes minimal discomfort [4, 5]. However, unlike histological diagnosis by CNB, FNAB only evaluates the morphology and characteristics of cells, thereby providing limited pathological insights and occasionally yielding false‐positive or false‐negative results [4]. In a meta‐analysis of 12 studies (including 1802 samples) that compared the diagnostic accuracy of FNAB with that of CNB for breast tumors, FNAB demonstrated a sensitivity of 74% (95% confidence interval 72–77) and a specificity of 96% (95% confidence interval 94–98) [3, 6, 7, 8]. Another report mentioned a high insufficiency/inadequacy rate of 17.7% with FNAB, which was attributed in part to the masses being too small for adequate collection of cells [9]. Other potential reasons for insufficient/inadequate samples include desiccation of cells and poor fixation [10]. Although the recently devised International Academy of Cytology Yokohama system is now used for breast FNAB with consistent reporting methods and clear definitions for each category [11, 12], FNAB does not guarantee absolute accuracy or definitively exclude cancer cells from breast tissue samples, even when they are absent. For initial evaluation of breast tumors, a comprehensive approach known as “triple assessment” is recommended, which involves clinical examination, breast imaging (mammography and/or ultrasound), and pathological evaluation [5]. Obtaining accurate results with FNAB also requires specialists to have appropriate technical skills and experience [13, 14].

We have developed a “click‐to‐sense” (CTS) probe that detects acrolein, a substance released from cancer cells under oxidative stress that can be visualized by fluorescence microscopy [15, 16]. Using live tissue samples resected from patients with breast cancer, Tanei et al. and Kubo et al. demonstrated that this CTS probe can rapidly, selectively, and sensitively differentiate between a cancerous breast lesion and a normal glandular or benign proliferative breast lesion and requires staining of live tissue for only 5 min [17, 18]. The CTS probe is an “in‐cell” reactivity probe for detection of acrolein by fluorescence based on an acrolein/azide click reaction and can visualize in detail the morphology of the various types of cancer cells in which acrolein accumulates [19]. Therefore, the CTS assay has potential for rapid cytopathological assessment of live cells obtained via FNAB. Although there have been reports of the ability of a fluorescent probe to distinguish between living cancer cells and normal cultured cells in vitro [20, 21, 22], there have been no reports on use of this novel diagnostic approach in live cells from patients.

In this study, we developed a CTS assay using live cells obtained for breast tumors via FNAB and compared its diagnostic accuracy with that of PAP staining of live tissue obtained from the same lesion by histopathological examination of permanent sections. The aim of this research was to determine whether the CTS assay can accurately distinguish between malignant and benign/normal breast tissues obtained by FNAB.

## 2 Materials and Methods

### 2.1 Patients and Breast Tumors

The study included 126 breast tissue samples from 89 patients who were retrospectively identified to have undergone benign tumorectomy (63 samples), or lumpectomy or mastectomy for primary breast cancer without neoadjuvant systemic therapy (63 samples) at Osaka University Hospital between March 2022 and January 2024 (Table 1). Two benign tumors were resected in one patient, and three benign tumors were resected at the same time as mastectomy for breast cancer. Thirty‐three normal breast gland tissue samples were obtained from distant breast tissue in the patients with breast cancer who underwent mastectomy. In total, FNAB examinations were performed for 126 breast tissue samples (63 breast cancers, 30 benign breast tumors, and 33 normal breast glands) and subjected to both the CTS assay and PAP staining. The breast tissues were fixed in 10% buffered formalin, embedded in paraffin, and diagnosed by histopathological examination of permanent sections (PS analysis).

**TABLE 1:** Histopathological characteristics of breast lesions.

	*N*			*N*	
	*n*	%		*n*	%
*Malignant*	63		*Benign/Normal*	63	
Ducal carcinoma in situ	9	14.3	Fibroadenoma	8	12.7
Low grade	3		Usual ductal hyperplasia	8	12.7
Intermediate grade	3		Intraductal papilloma	6	9.5
High grade	3		Mastopathy	3	4.8
Invasive ductal carcinoma	43	68.3	Phyllodes tumor	3	4.8
Histological grade 1	12		Adenomyoepithelioma	1	1.6
Histological grade 2	32		Mucocele‐like leision	1	1.6
Histological grade 3	10		Normal breast tissue	33	52.4
Mucinous carcinoma	5	7.9			
Invasive lobular carcinoma	3	4.8			
Encapsulated papillary carcinoma with invasion	1	1.6			
Apocrine carcinoma	1	1.6			
Invasive micropapillary carcinoma	1	1.6			

### 2.2 Reagents

The CTS probe was synthesized using a method previously documented in the literature [16]. Four micrograms of the CTS probe were dissolved in 1 μL of dimethyl sulfoxide. Following this, 4 μL of Hoechst 33342 dye (Dojindo, Kumamoto, Japan), 4 μL of Hoechst 33258 dye (Dojindo), and 490 μL of phosphate‐buffered saline were added to prepare a CTS probe solution with a final concentration of 12 μM. For each analysis, 25 μL of the CTS probe was mixed with 25 μL of the encapsulating agent, ProLong Live Antifade Reagent (Thermo Fisher Scientific, Fresno, CA, USA), and placed onto a glass slide. The mechanism by which acrolein can be visualized in cancer cells using the CTS probe (see Figure S1) was detailed in our previous work [19], allowing for the labeling and imaging of cancerous tissue at the cellular level.

### 2.3 CTS Assay

**FIGURE 1:**
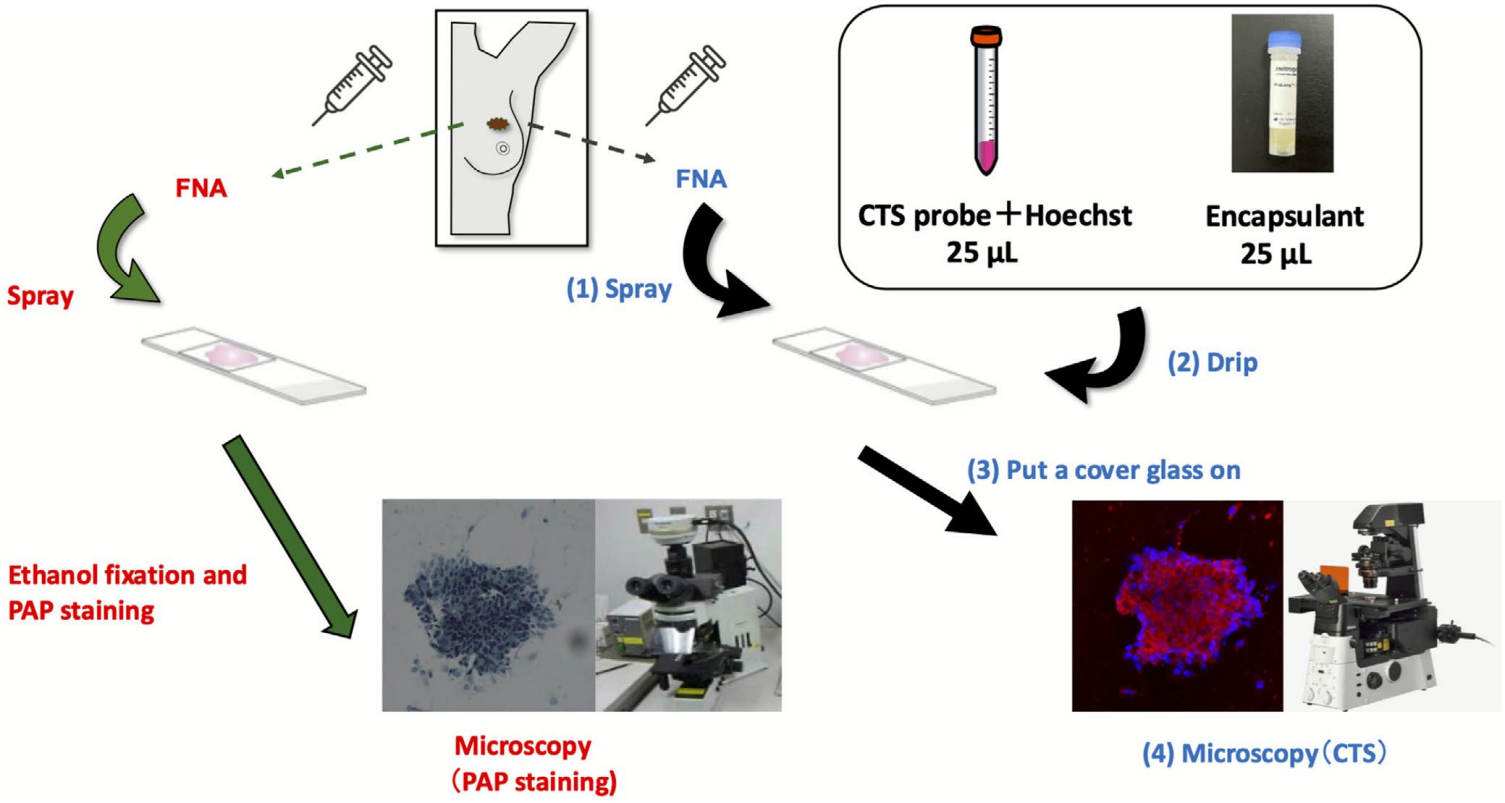
Schematic diagram showing the experimental procedures. The CTS assay is shown in blue and the PAP stain analysis in red. Two FNA biopsies were performed for each lesion obtained from a live breast tissue sample. In the CTS assay, the cells were spread onto a glass slide (1), followed by immediate application of the CTS probe containing Hoechst dye and encapsulating agent (2). Finally, the slides were sealed with a glass cover slip (3) and imaged using a fluorescence microscope (4). CTS, click‐to‐sense; FNA, fine‐needle aspiration; PAP, Papanicolaou.

We used the CTS assay to analyze 126 breast tissue samples as well as fluorescence microscopy analysis using both CTS probe fluorescence staining (red) and Hoechst 33342 and 33258 fluorescence staining (blue) (Figure 1). Each live breast tissue sample was obtained by FNAB using a 22‐G needle and a 10‐mL syringe. The contents of the syringe were then placed on a glass slide, after which 50 μL of the CTS probe with Hoechst dye and encapsulating agent were immediately dripped onto the slide. Finally, a glass coverslip was applied and the slide was imaged using a fluorescence microscope. Whole‐slide fluorescence images with a range of 2 × 1 cm were captured in a low‐power field (40×). Images of cell clusters stained red by the CTS probe were also captured in high‐power fields (200×, range, 5 mm × 5 mm/400×, range, 2.5 mm × 2.5 mm) for morphological examination (Figure S2). The mean time required for diagnosis using the CTS assay was 5 min per tissue sample, and only one person was required to perform the assay.

### 2.4 Analysis of CTS Images

**FIGURE 2:**
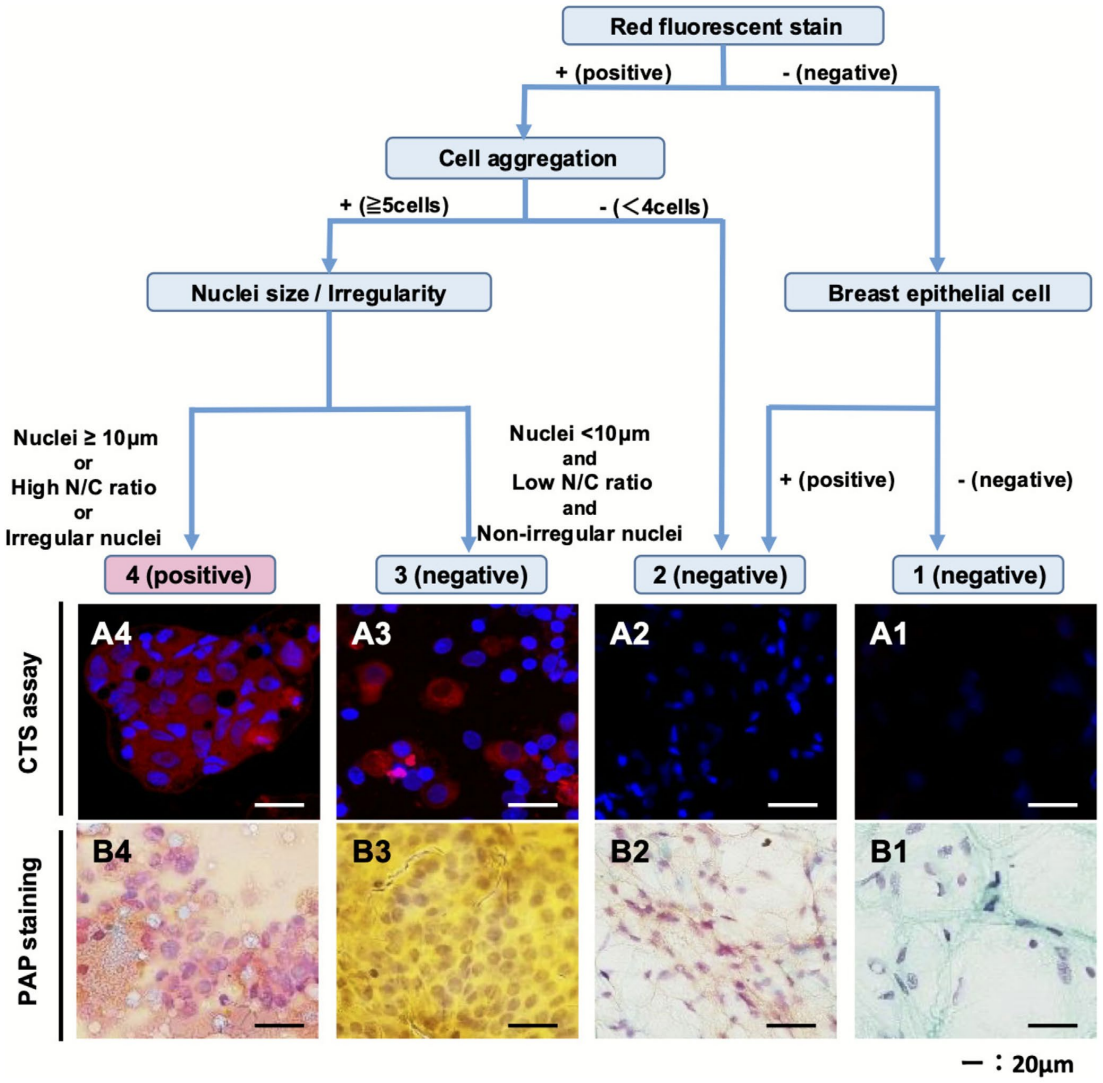
Flow chart showing the diagnostic criteria for the CTS assay with CTS and PAP stained images. In the diagnostic criteria for the CTS assay, category 4 (positive) is diagnosed by cells with red fluorescence in the cytoplasm, appearing as ≥ 5 aggregated fluorescent cells with nuclei that are enlarged (≥ 10 μm) or irregular in shape. Both representative fluorescence microscopic images for the CTS assay and PAP stain analysis demonstrated invasive ductal carcinoma (category 4), fibroadenoma (category 2–3), a mucocele‐like lesion (category 2), and normal breast tissue (category 1). CTS, click‐to‐sense; N/C, nuclear/cytoplasmic; PAP, Papanicolaou.

CTS images were analyzed in accordance with the diagnostic criteria outlined in the CTS assay flow chart (Figure 2) and categorized into four groups. Representative fluorescence and PAP stained images for each category are shown in Figure 2. Category 4 was diagnosed as malignant (positive) and categories 1–3 as benign or normal (negative). In Figure 2, representative fluorescence microscopy images obtained by the CTS assay and by PAP staining revealed invasive ductal carcinoma (category 4), fibroadenoma (category 2–3), a mucocele‐like lesion (category 2), and normal breast tissue (category 1). PAP staining identified the invasive ductal carcinoma as malignant (Figure 2B4) and the fibroadenoma and normal breast tissue as benign (Figure 2B1–B3).

In category 4, fluorescent aggregated cells (≥ 5) were red in the cytoplasm with enlarged nuclei (≥ 10 μm) and had a high nuclear/cytoplasmic (N/C) ratio and irregular nucleus. We investigated the major axis of the nuclei of cells stained with Hoechst dye (blue) (Figure 2A4). The criterion for defining cells with enlarged nuclei (≥ 10 μm) as malignant was based on an examination of the major nuclear axis of 20 cancer cells and 20 normal or benign ductal epithelial cells stained with Hoechst dye (blue) in which the CTS assay established 10 μm as the threshold for differentiation between malignant and benign/normal cells (Figure S3).

In category 3, although fluorescent aggregated cells (≥ 5) were red in the cytoplasm, they had a low nuclear cytoplasmic (N/C) ratio and smaller nuclei, were < 10 μm in size, and had a regular shape (Figure 2A3). In category 2, epithelial cells without fluorescent aggregated cells (< 4) were detected (Figure 2A2). In category 1, the CTS assay did not identify any epithelial cells with cytoplasmic staining (Figure 2A1).

Three blinded diagnosticians working independently visually evaluated each CTS image to determine whether it was positive or negative. CTS images were deemed to be positive when diagnosed by two or three diagnosticians as positive using the CTS assay and to be negative when diagnosed as positive by zero or one diagnostician. The diagnosticians registered their findings in the Research Electronic Data Capture (Redcap) system hosted at Osaka University using a HIPAA‐compliant secure web application [23]. Access to servers and systems is restricted by user accounts and passwords, and a full audit trail is recorded.

### 2.5 Papanicolaou Staining

We performed FNAB twice for each lesion. The second samples were fixed in ethanol and subjected to PAP staining, after which they were compared with the samples subjected to the CTS assay (Figure 1). The PAP staining protocol was performed following the same procedure as that used in the Pathology Department at Osaka University. PAP staining was interpreted by skilled experienced cytopathologists and specialists using International Academy of Cytology Yokohama system‐compliant categories.

### 2.6 Measurement of Fluorescence Intensity of the CTS Assay

Regions of cellular aggregation were excised from the CTS images and analyzed by Nikon NIS‐Elements image analysis software with 200× magnification. The median fluorescence intensity of the areas that were stained red was measured in each cell.

### 2.7 Statistical Analysis

The diagnostic accuracy of the CTS assay was compared with that of PAP staining by PS analysis. The McNemar test was used to assess the relationship between the results of the CTS assay and those for PAP staining. The Mann–Whitney test was used to compare the fluorescence intensity of the CTS assay between malignant and benign/normal breast tissue. All statistical analyses were performed using R version 4.0.2 (R Foundation for Statistical Computing, Vienna, Austria). A *p*‐value < 0.05 was deemed to be statistically significant.

## 3 Results

### 3.1 Microscopic Fluorescence Images of the CTS Assay

**FIGURE 3:**
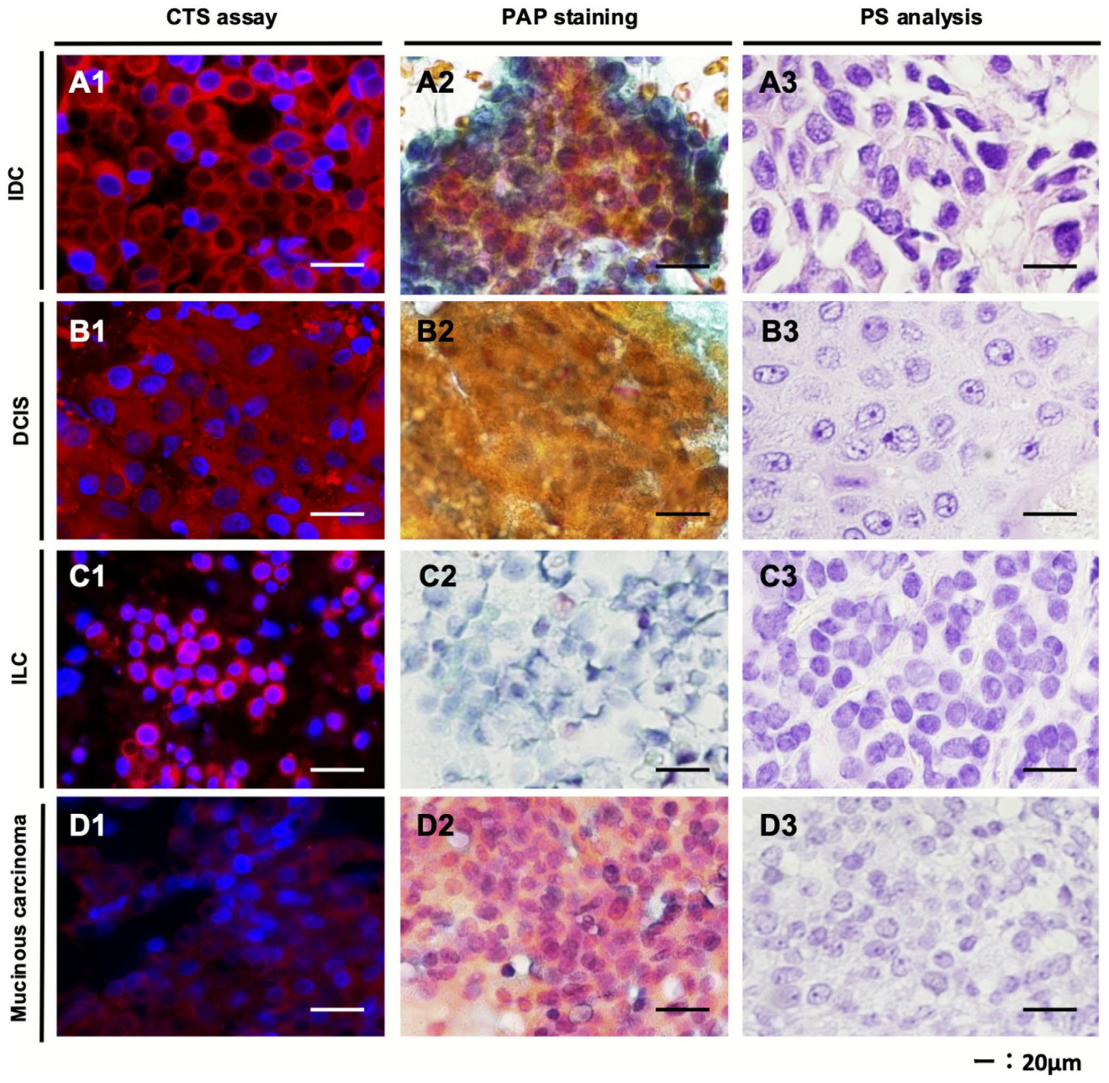
Representative images of CTS assay‐positive samples. Images of positive samples represented by the CTS assay (A1–D1), PAP stain analysis (A2–D2), and histopathological images of permanent sections (A3–D3). Representative images are shown for breast tissue samples positive for invasive ductal cancer, ductal carcinoma in situ, invasive lobular carcinoma, and mucinous carcinoma. CTS, click‐to‐sense; PAP, Papanicolaou; PS, permanent section.

When using the CTS assay, cells were identified from whole‐slide fluorescence images, and images of cell clusters stained red by the CTS probe were observed under high magnification for examination of cell morphology (Figure S2). Representative CTS assay‐positive and PAP staining‐positive images from invasive ductal cancer, ductal carcinoma in situ, invasive lobular carcinoma, or mucinous carcinoma are shown in Figure 3. The cytoplasm of malignant cells was stained with TAMRA fluorescence dye derived from the CTS probe (red), and the nuclei of these cells were stained with Hoechst dye (blue). The malignant cells clearly showed morphological features similar to those observed with PAP staining and PS analysis.

**FIGURE 4:**
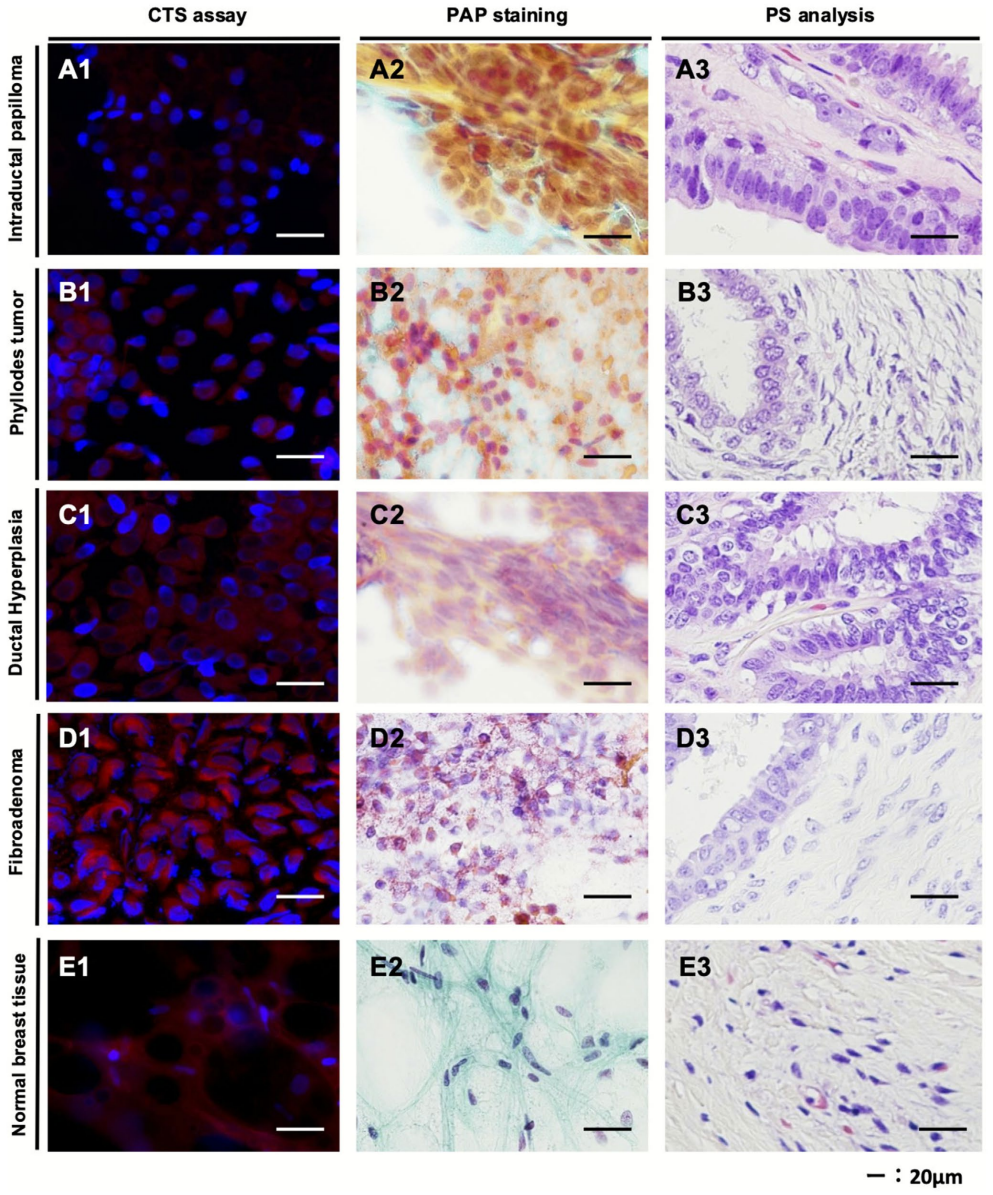
Representative images of CTS assay‐negative samples. Images of negative samples represented by the CTS assay (A1–E1), PAP stain analysis (A2–E2), and histopathological images of permanent sections (A3–E3). Representative images are shown for intraductal papilloma (A1–A3), phyllodes tumor (B1–B3), ductal hyperplasia (C1–C3), fibroadenoma (D1–D3), and normal breast tissue (E1–E3). CTS, click‐to‐sense; PAP, Papanicolaou; PS, permanent section.

Representative CTS assay‐negative and PAP staining‐negative images from benign lesions and normal breast tissue are shown in Figure 4. CTS images of epithelial cells from benign lesions, such as intraductal papilloma, phyllodes tumor, ductal hyperplasia, and fibroadenoma, detected minimal or weak red cytoplasm that was less intense than that of malignant cells (Figure 4A1–D1). CTS images of normal breast tissues were detected with only nuclei of no red cytoplasmic cells (Figure 4E1). There were clear differences in morphological features and boundaries between malignant and benign/normal cells.

### 3.2 Comparison of the CTS Assay and PAP Staining With PS Analysis

**FIGURE 5:**
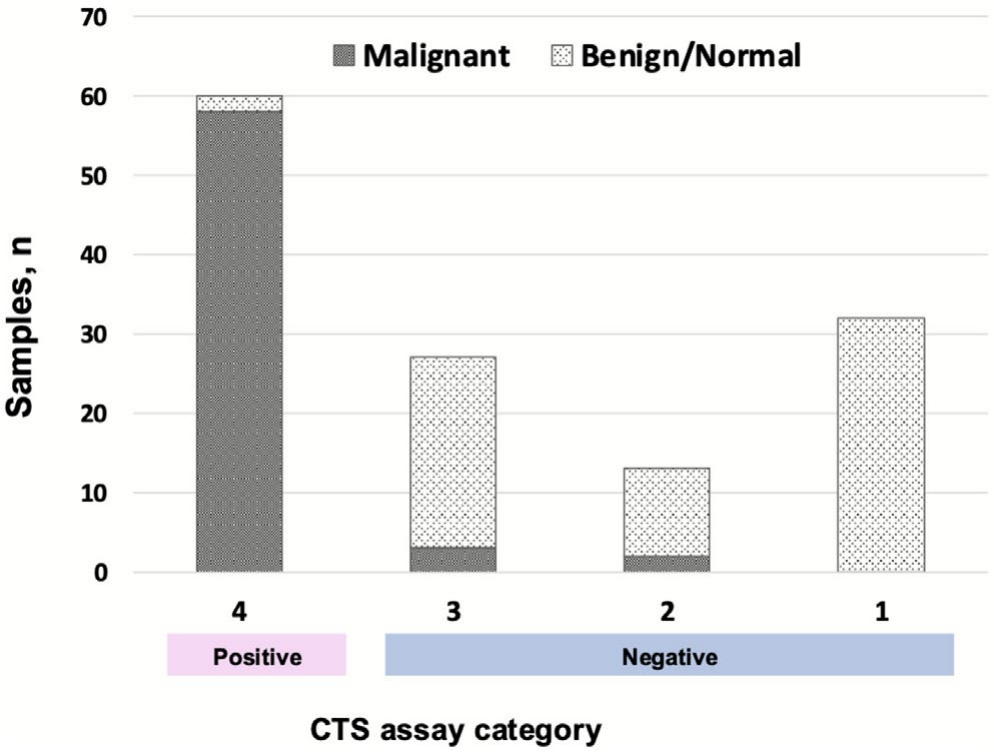
Correlation of pathological results with each of the four categories in the CTS assay. The judgment chosen by the majority of the three diagnosticians was adopted. The diagnostic accuracy of the CTS assay was as high as 94.4% with a sensitivity of 92.1% and a specificity of 96.8% in all samples. The accuracy of the CTS assay was 96.7% (58/60 samples) for category 4 (positive), 88.9% (24/27) for category 3 (negative), 84.6% (11/13) for category 2 (negative), and 100% (26/26) for category 1 (negative). CTS, click‐to‐sense.

The results of the CTS assay and PAP staining analysis are compared with those of the PS analysis in Table 2. The results of the CTS assay and PAP staining are shown according to histological type in Table S1. As shown in Figure 5, 60 samples were classified as positive (category 4) by the CTS assay, 58 of the 60 samples were diagnosed as malignant by PS analysis, and two were classified as benign/normal (accuracy, 96.7%). Twenty‐seven samples were classified as category 3 (benign/normal, *n* = 24; malignant, *n* = 3; accuracy, 88.9%). Thirteen samples were classified as category 2 (benign/normal, *n* = 11; malignant, *n* = 2; accuracy, 84.6%). All 26 samples classified as category 1 were benign/normal on PS analysis (accuracy, 100%). PAP staining had an insufficiency/inadequacy rate of 4.0% (5/126 samples). In contrast, none of the 126 samples were insufficient/inadequate for the CTS assay. Finally, the diagnostic accuracy of the CTS assay was 94.4% with a sensitivity of 92.1% and a specificity of 96.8%. Meanwhile, the diagnostic accuracy of the PAP staining was 94.2% with a sensitivity of 98.4% and a specificity of 89.8% after exclusion of insufficient/inadequate samples (Table 2). There was no significant difference in diagnostic accuracy between the CTS assay and PAP staining analysis (*p* = 0.8, McNemar's test).

**TABLE 2:** Comparison of the results of the CTS assay and PAP staining analysis with those of the PS analysis.

		PS analysis		Accuracy rate	Insufficient/Inadequate rate
		Malignant	Benign/Normal		
CTS assay	Positive	58	2	94.4%	
	Negative	5	61		
	Insufficient/Inadequate	0	0		0%
PAP staining	Malignant/Suspicious	61	6	94.2%	
	Atypical	0	2		
	Benign	1	51		
	Insufficient/Inadequate	1	4		4%

Abbreviations: CTS, click‐to‐sense; PS, permanent section.

### 3.3 False‐Positive and False‐Negative CTS Assay Samples

There were two false‐positive CTS assay samples, one of which was intraductal papilloma and the other was fibroadenoma with epithelial cells that stained faintly red in the CTS assay. However, there were few red‐stained cells without nuclear enlargement (Figure S4). The CTS assay also yielded five false‐negative results (invasive ductal cancer, *n* = 2; invasive lobular carcinoma, *n* = 1; ductal carcinoma in situ, *n* = 1; mucinous carcinoma, *n* = 1; Figure S5). Cells in the two invasive ductal cancer samples and in the invasive lobular carcinoma sample strongly stained red in the CTS assay. However, these cells had small, non‐irregular nuclei, leading to misclassification as negative.

**FIGURE 6:**
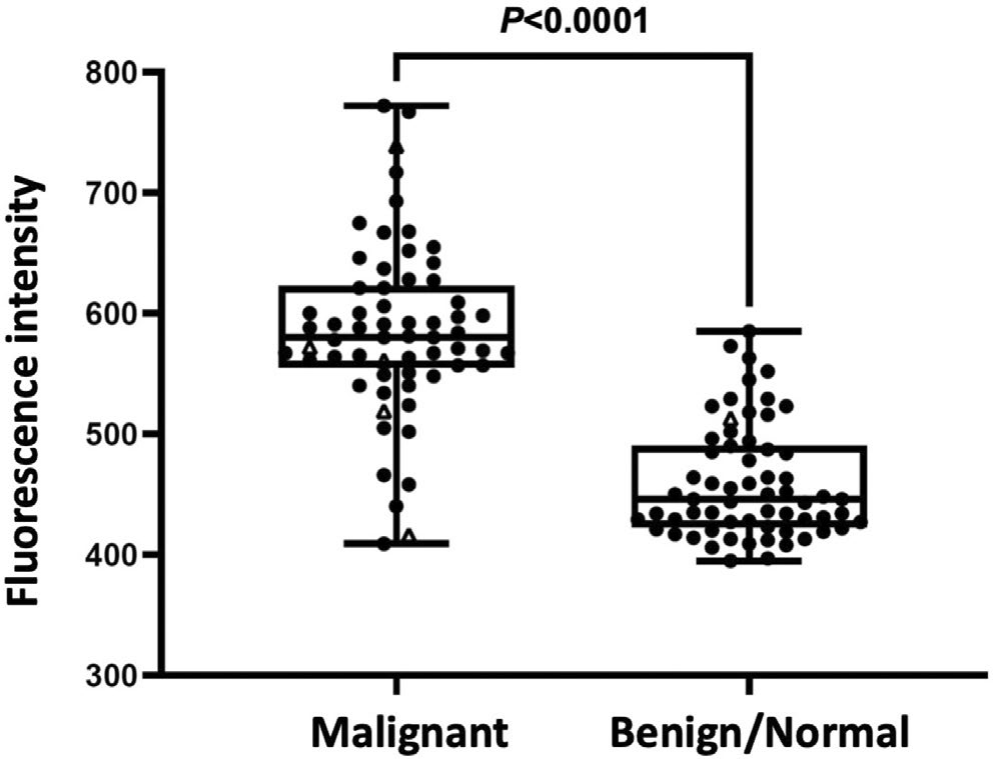
Fluorescence intensity in malignant and benign/normal breast cells stained red by the CTS probe. Red fluorescence intensity was measured in regions of cell aggregation on CTS images for 126 samples with 200× magnification. Red staining of these images was much stronger in breast cancer cells than in cells from benign/normal breast tissue. Δ, misclassification by the CTS assay. A statistically significant difference was observed between malignant and benign/normal breast tissues (*p* < 0.0001). CTS, click‐to‐sense.

Red fluorescence intensity was measured in regions of cell aggregation on CTS images of 126 samples with 200× magnification. We found that red staining of CTS images was much stronger in breast cancer cells than in cells from benign/normal breast tissue. There were significant differences in red fluorescence intensity on CTS images for malignant cells among each group (Mann–Whitney test). There was a statistically significant difference in fluorescence intensity between malignant and benign/normal breast tissue (*p* < 0.0001, Figure 6).

## 4 Discussion

We have previously found the CTS assay to be effective for rapid differential diagnosis of malignant and benign/normal breast lesions, suggesting a potential application for this assay in FNAB of breast tumors. In this study, we compared the accuracy of the CTS assay with that of standard PAP staining by observing cells obtained from malignant and benign tumors and from normal breast tissues by FNAB. We performed FNAB twice on the live tissue samples and evaluated each slide using the CTS assay and PAP staining. We also standardized the morphological examination visualized by the CTS assay and developed a flow chart showing the diagnostic criteria that should be used when performing this assay (Figure 2). Finally, we were able to obtain a very high accuracy rate of 94.4% (119/126 samples) between the CTS assay and PS analysis when performed in a blinded manner, which was comparable with the rate of 94.2% (114/121 samples) between the PAP staining and PS analyses. In particular, sensitivity was lower and specificity was higher for the CTS assay than for the PAP staining analysis. The number of individuals diagnosed as positive by three independent diagnosticians is shown in Table S2. A significant proportion (57%) of CTS‐positive samples were consistently diagnosed as positive by all three diagnosticians, while the majority (80%) of CTS‐negative samples were consistently diagnosed as negative by the same three diagnosticians.

In this study, the CTS assay achieved a negative predictive value (NPV) of 96.8%, compared to 89.8% achieved by PAP staining. This finding demonstrated to the high reliability of the CTS assay in accurately accurate diagnosis of benign lesions. The CTS assay holds significant potential for substantially reducing the risk of overlooking malignant lesions in negative diagnoses. In clinical settings with a high prevalence of benign lesions, the superior NPV of the CTS assay have advantage of offering the potential to minimize follow‐up assessments and invasive procedures.

However, the positive predictive value (PPV) of the CTS assay was 92.1%, whereas that of PAP staining was 98.4%. A comparison of the CTS assay and PAP staining results revealed an overall concordance rate of 90.1% (Table S3). The CTS assay and PAP staining often resulted in different outcomes, with only one case, a fibroadenoma, being misclassified by both methods. In this case, both CTS and PAP staining incorrectly diagnosed the sample as positive. For all other cases, even if one method produced an incorrect result, the other method provided an accurate diagnosis. Importantly, the CTS assay was able to correctly identify cases that were falsely classified as positive by PAP staining. This suggests that the CTS assay could potentially be used as an aid to PAP staining in diagnostic evaluation.

We encountered two false‐positives and five false‐negatives when using the CTS assay. The two false‐positives were intraductal papilloma and fibroadenoma, which showed lightly red‐stained epithelial cells in the CTS assay (Figure S4). Two of the three diagnosticians deemed these benign lesions to be positive, even though there were usually few red‐stained cells without nuclear enlargement, indicating the presence of benign cells with high oxidative stress stained in red by the CTS assay. The five false‐negatives included two samples of invasive ductal cancer, one of invasive lobular carcinoma, one of ductal carcinoma in situ, and one of mucinous carcinoma. Two of the three diagnosticians diagnosed these four malignancies as negative. The sample with ductal carcinoma in situ showed few cancer cells on PAP staining and was diagnosed as a single‐duct ductal carcinoma in situ by PS analysis, and the CTS assay result was a consequence of poor cell sampling in FNAB (Figure S5A1–A3). Although there were cells that strongly stained red in the CTS assay for two samples of invasive ductal cancer and one of invasive lobular carcinoma, these cells were small and had non‐irregular nuclei, which led to misclassification as negative (Figure S5B1–B3, C1–C3, D1–D3). The mucinous carcinoma sample showed weak staining and had small nuclei (Figure S5E1–E3), leading to a benign diagnosis because the CTS reagent became diluted after excessive application of liquid, which reduced the intensity of red fluorescence.

To evaluate the potential reduction of false‐positives and false‐negatives, we measured the nuclear size by their major axes selecting five representative nuclei stained blue with Hoechst of all 126 samples (Figure S6). The results demonstrated a significant difference in nuclear size between malignant and benign/normal samples, as well as Figure S3. False‐positives frequently exhibited larger nuclei, whereas false‐negatives predominantly displayed smaller nuclei. More precise measurements of nuclear size might contribute to improving the diagnostic accuracy of the CTS.

Furthermore, none of the samples were considered insufficient/inadequate for the CTS assay, but 4% were found to be insufficient/inadequate for PAP staining analysis. This difference may be explained by the fact that unlike PAP staining, the CTS assay does not include fixing in ethanol, which is associated with desiccation of cells and poor fixation.

Use of the CTS assay for FNAB requires only simple techniques, namely, application of the CTS probe, Hoechst dye, and encapsulating agent to a glass slide containing aspirated cells followed by cover‐slipping. In contrast, the PAP staining process is complex and must be performed by an expert cytopathologist. The CTS assay has the advantages of high specificity, no risk of insufficient/inadequate sampling, and being simple to perform, time‐saving, and less labor‐intensive in comparison with PAP staining analysis. In clinical practice, it takes at least a few days to report the PAP staining results. In contrast, the results of the CTS assay are available after 5 min. Rapid onsite evaluation systems can now triage FNAB samples using ultrasound guidance, although a cytopathologist is still needed for FNAB analysis [24, 25, 26]. Breast FNAB and PAP staining analysis require specific training in technique and slide interpretation for accurate reporting and involves a steep learning curve [14]. Meanwhile, the CTS assay can visualize cancer cells without the need for a pathologist. The workload for this assay entails checking the fluorescence images for red‐stained cells with irregular nuclear contours or nuclear enlargement. This study demonstrates that the CTS assay makes a significant contribution to cytopathological diagnosis of live cancer cells and normal cells obtained from patients, despite previous reports indicating that this assay is only useful for diagnosis of cultured cells in vitro [20, 21, 22].

However, the CTS assay has some limitations. The first limitation of the CTS assay, as shown in the diagnostic flowchart in Figure 2, is that red staining alone makes it difficult to diagnose malignancy. In the diagnosis of benign samples, the absence of red staining allows for a clear benign classification, reducing the number of false positives. However, in cases where nuclear morphological abnormalities are mild or moderate, false negatives may occur, as observed in this study. Second, unlike PAP staining, the CTS assay does not provide details on internal nuclear structure, and cannot identify histological types. This restricts the ability to offer the detailed pathological information needed for thorough diagnosis and treatment planning. The third limitation is the lack of well‐defined intermediate diagnostic categories, such as “atypical” or “indeterminate,” of PAP staining for determining follow‐up assessments and invasive procedures. In the CTS assay, Category 3 includes a wider range of samples than the intermediate categories in PAP staining. In the future, Category 3 applying stricter criteria and creating more refined classification, the CTS assay might potentially achieve a similar level of diagnostic detail as PAP staining. The fourth limitation of the CTS assay is that it cannot be preserved for a long time, as fluorescence will degrade. Digitalization may be a way to preserve the data. The original fluorescence on the slides can hardly be double‐checked afterwards if there is any diagnostic dispute later. The last limitation is to include its single‐center design and the limited sample size. The potential utility of our method for FNAB using the CTS assay requires assessment in larger multicenter clinical trials.

Research is underway at our center to develop an artificial intelligence (AI)‐assisted diagnostic imaging system for use with the CTS assay, which may represent an advance on the cytopathological diagnostic methods available. We are currently conducting a collaborative research project with the Graduate School of Information Science and Technology at Osaka University in the hope of developing a program that uses AI‐driven techniques for diagnosis of breast cancer on fluorescence images obtained using the CTS assay. Recent advances in AI are transforming the diagnosis of breast cancer on hematoxylin–eosin‐stained slides, significantly improving the accuracy, efficiency, and consistency of PS analysis. Several innovative AI‐based tools are commercially available, offering pathologists and oncologists valuable support when diagnosing breast cancer [27]. The first use of AI in a pathology laboratory was in cytopathology when a computer‐assisted PAP staining screening method was created [28]. AI technologies have the potential to completely transform cytopathology. In a cervical screening study, Kurita et al. demonstrated that AI could classify images of liquid‐based cervical cytology samples as malignant or normal and that AI would allow cytologists or cytopathologists to obtain support from their counterparts worldwide [29]. We speculate that the CTS assay could potentially be applied to cytology for other cancers, such as cervical carcinoma, and we are presently conducting a clinical trial of the CTS assay for cervical cytology of PAP smears. In summary, the CTS assay offers diagnostic accuracy comparable with that of PAP staining analysis in FNAB of breast tumors. It is simpler and less labor‐intensive and time‐consuming than PAP staining analysis. While the assay currently has certain limitations, we believe it has the potential to be further refined and to contribute to the establishment of a novel diagnostic approach in cytopathology for FNAB in the future. Currently, the CTS assay is evaluated visually. However, near‐future technology, diagnosis may be possible using AI‐based images, which would be a significant advance in cytopathological diagnostic methodology. We also believe that there is potential for application of cytopathological diagnosis to other cancerous lesions in the future.

We have shown that the CTS assay is as accurate as PAP staining in FNAB for breast tumors. This suggests that it has the potential to be used as an aid in diagnosis, and with further refinement, it could eventually be established a new diagnostic method in cytopathology in the future. The insufficiency/inadequacy rate is lower for the CTS assay than for PAP staining. Furthermore, the CTS assay is simpler, less labor‐intensive, and less time‐consuming to perform.

## Author Contributions

**Yuri Kitahara:** resources, visualization, investigation, writing – original draft. **Tomonori Tanei:** conceptualization, methodology, investigation, writing – original draft, writing – review and editing, visualization, supervision, project administration, funding acquisition. **Takaaki Hatano:** resources, formal analysis, writing – review and editing. **Ambara R. Pradipta:** investigation, resources, funding acquisition writing – review and editing. **Koji Morimoto:** visualization, writing – review and editing. **Tadasuke Nagatomo:** resources, visualization. **Kaori Abe:** writing – review and editing. **Nanae Masunaga:** writing – review and editing. **Chieko Mishima:** writing – review and editing. **Tetsuhiro Yoshinami:** writing – review and editing. **Masami Tsukabe:** writing – review and editing. **Yoshiaki Sota:** software, formal analysis, writing – review and editing. **Tomohiro Miyake:** writing – review and editing, visualization. **Masafumi Shimoda:** term, writing – review and editing. **Yuichi Motoyama:** resources, visualization. **Eiichi Morii:** project administration. **Katsunori Tanaka:** methodology, funding acquisition. **Kenzo Shimazu:** project administration.

## Funding

This work was supported by JSPS KAKENHI (Grant Numbers JP22K08709, JP24K11762, and JP24K01625), AMED (Grant Number JP22ck0106783), AMED FORCE, and the Canon Foundation.

## Ethics Statement

This study was conducted in accordance with the ethical principles outlined in the Declaration of Helsinki and was approved by the Medical Ethics Committee of Osaka University (Approval Number: 20197). All procedures performed in this study involving human participants were in compliance with recognized standards, including the US Federal Policy for the Protection of Human Subjects and the International Council for Harmonization Guidelines for Good Clinical Practice (ICH‐GCP). Informed consent was obtained from all participants via an opt‐out approach, as detailed on the hospital's website.

## Conflicts of Interest

The authors declare no conflicts of interest.

## Supporting information


**Figure S1:** Synthesis of the CTS probe and the mechanism via which it visualizes acrolein. CTS, click‐to‐sense.

**Figure S2:** Fluorescence images captured in the CTS assay. Whole‐slide fluorescence images (area, 2 × 1 cm) captured at low magnification (40×). Images of cell clusters stained red by the CTS probe were also captured at high magnification (200×, area 5 mm × 5 mm/400×, area 2.5 mm × 2.5 mm) for morphological examination. CTS, click‐to‐sense.

**Figure S3:** The Major axis of nuclei. The major axes of 20 cancer cells and 20 normal ductal epithelial cells were measured in the CTS images. A threshold of 10 μm was established in the CTS assay to differentiate between malignant and benign/normal cells. CTS, click‐to‐sense.

**Figure S4:** Representative images of CTS assay false‐positive samples. CTS images were classified as positive by the CTS assay (A1–B1). On PAP stain analysis, A2 was classified as negative and B2 as positive. In contrast, the histopathological images (A3–E3) were diagnosed as intraductal papilloma (A1–A3) and fibroadenoma (B1–B3). CTS, click‐to‐sense; PAP, Papanicolaou; PS, permanent section.

**Figure S5:** Representative images of CTS assay false‐negative samples. CTS images were classified as negative by the CTS assay (A1–E1). On PAP stain analysis, A2–E2 was classified as positive. In contrast, histopathological images (A3–E3) were diagnosed as low‐grade DCIS (A1–A3), IDC (B1–B3, C1–C3), ILC (D1–D3), and mucinous carcinoma (E1–E3). CTS, click‐to‐sense; DCIS, ductal carcinoma in situ; IDC, invasive ductal carcinoma; ILC, invasive lobular carcinoma; PAP, Papanicolaou; PS, permanent section.

**Figure S6:** The Major axis of nuclei in the 126 CTS images. The major axes of nuclei of representative cells were measured in the 126 CTS images. Δ, misclassification by the CTS assay. A statistically significant difference was observed between malignant and benign/normal breast tissues (*p* < 0.0001).

**Table S1:** Results of the CTS assay and PAP staining analysis according to histological type.

**Table S2:** Number of samples diagnosed as positive by three diagnosticians working independently.

**Table S3:** Comparison of the CTS assay and PAP staining results.

## Data Availability

The data that support the findings of this study are available on request from the corresponding author. The data are not publicly available due to privacy or ethical restrictions.
