# Supplementary figures and images for: Rapid and Accurate Diagnosis of Breast Cancer by Fine‐Needle Aspiration Biopsy Using the “Click‐to‐Sense” Method
Source: Cancer Med. 2026 Feb 13;15(2):e71525. doi: 10.1002/cam4.71525 (PMC12903542; doi:10.1002/cam4.71525)

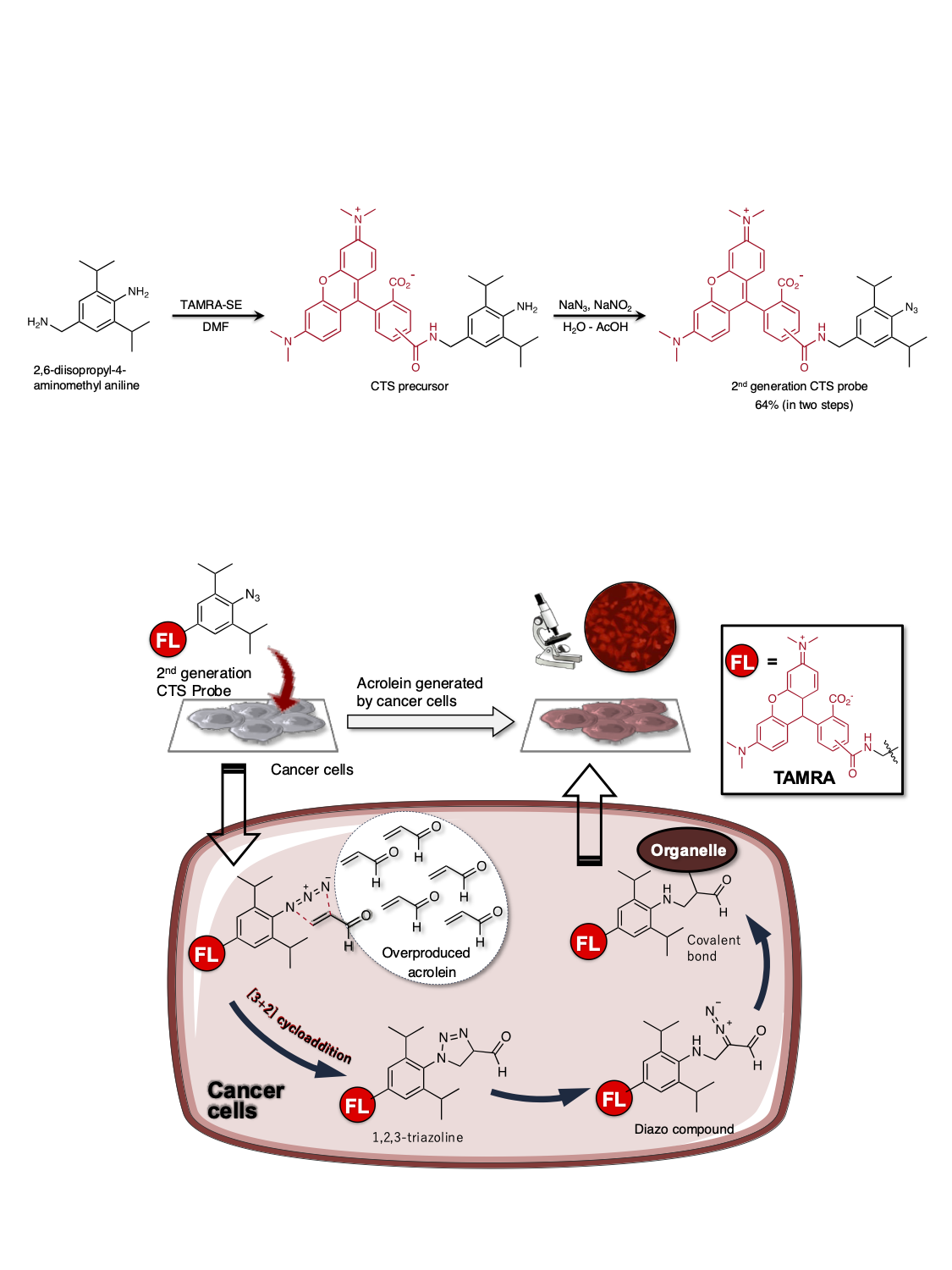

Supplement: Supplementary file 1 — Figure S1: Synthesis of the CTS probe and the mechanism via which it visualizes acrolein. CTS, click‐to‐sense. [file CAM4-15-e71525-s005.tiff]

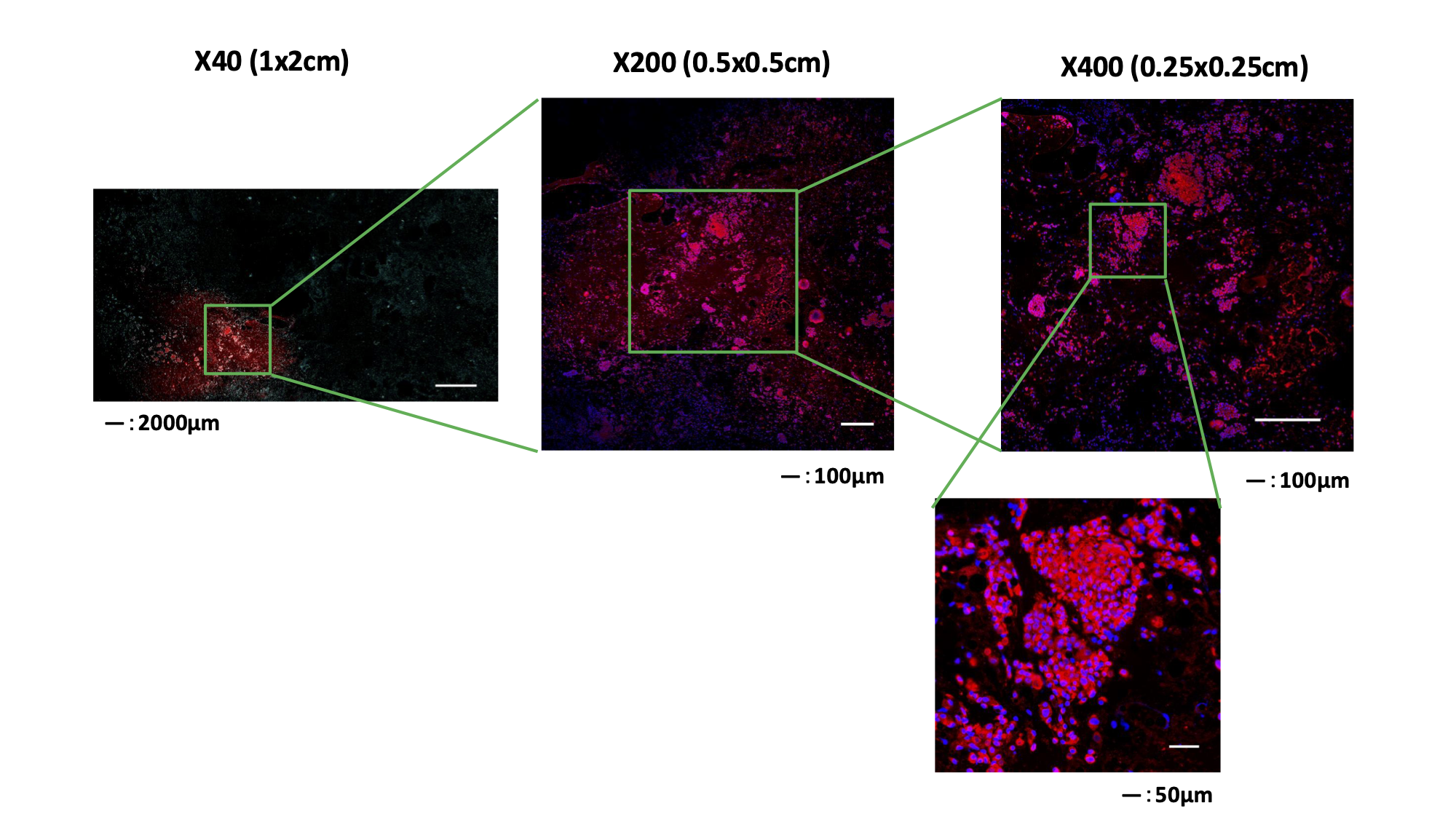

Supplement: Supplementary file 2 — Figure S2: Fluorescence images captured in the CTS assay. Whole‐slide fluorescence images (area, 2 × 1 cm) captured at low magnification (40×). Images of cell clusters stained red by the CTS probe were also captured at high magnification (200×, area 5 mm × 5 mm/400×, area 2.5 mm × 2.5 mm) for morphological examination. CTS, click‐to‐sense. [file CAM4-15-e71525-s004.tiff]

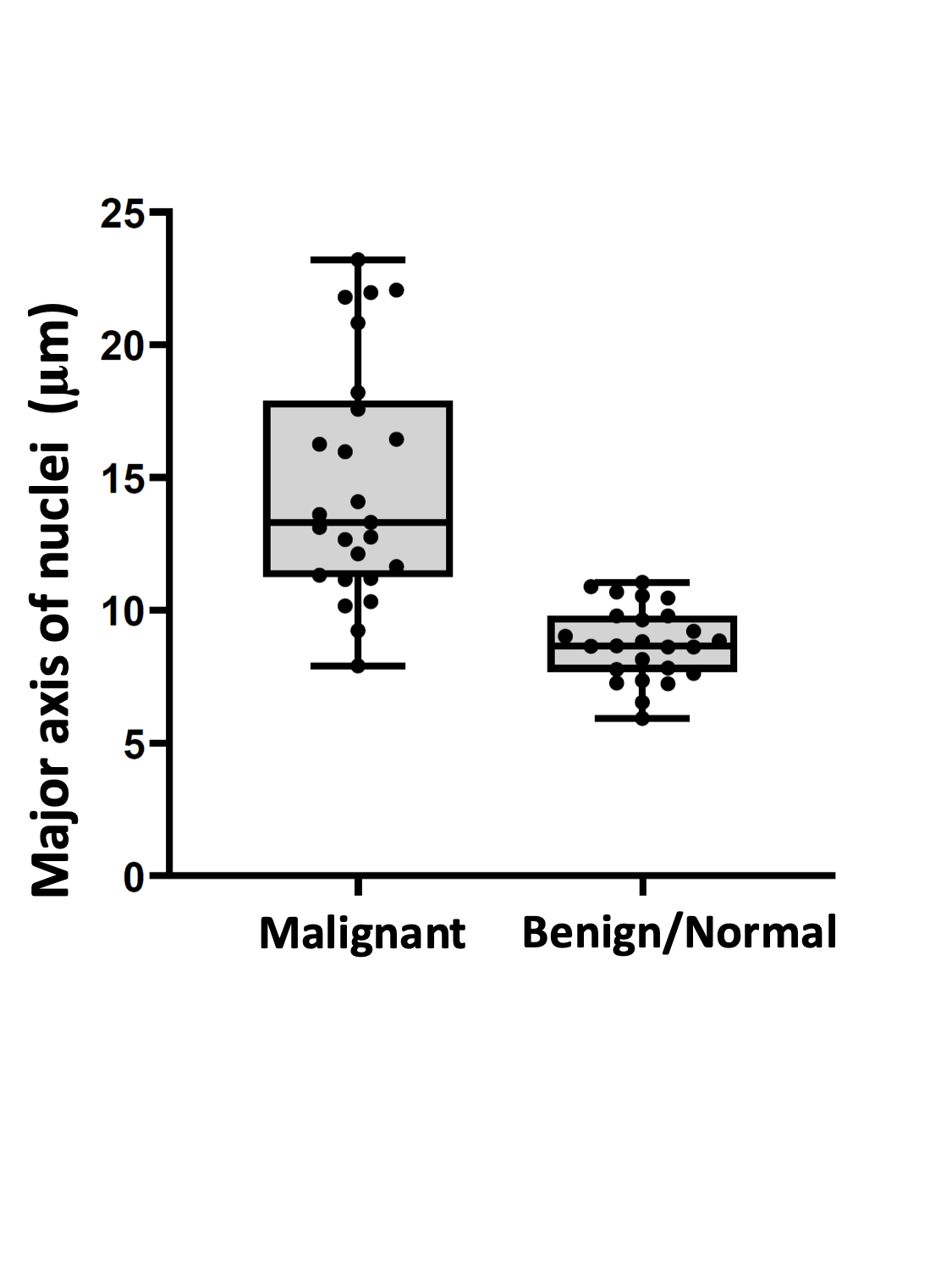

Supplement: Supplementary file 3 — Figure S3: The Major axis of nuclei. The major axes of 20 cancer cells and 20 normal ductal epithelial cells were measured in the CTS images. A threshold of 10 μm was established in the CTS assay to differentiate between malignant and benign/normal cells. CTS, click‐to‐sense. [file CAM4-15-e71525-s002.tiff]

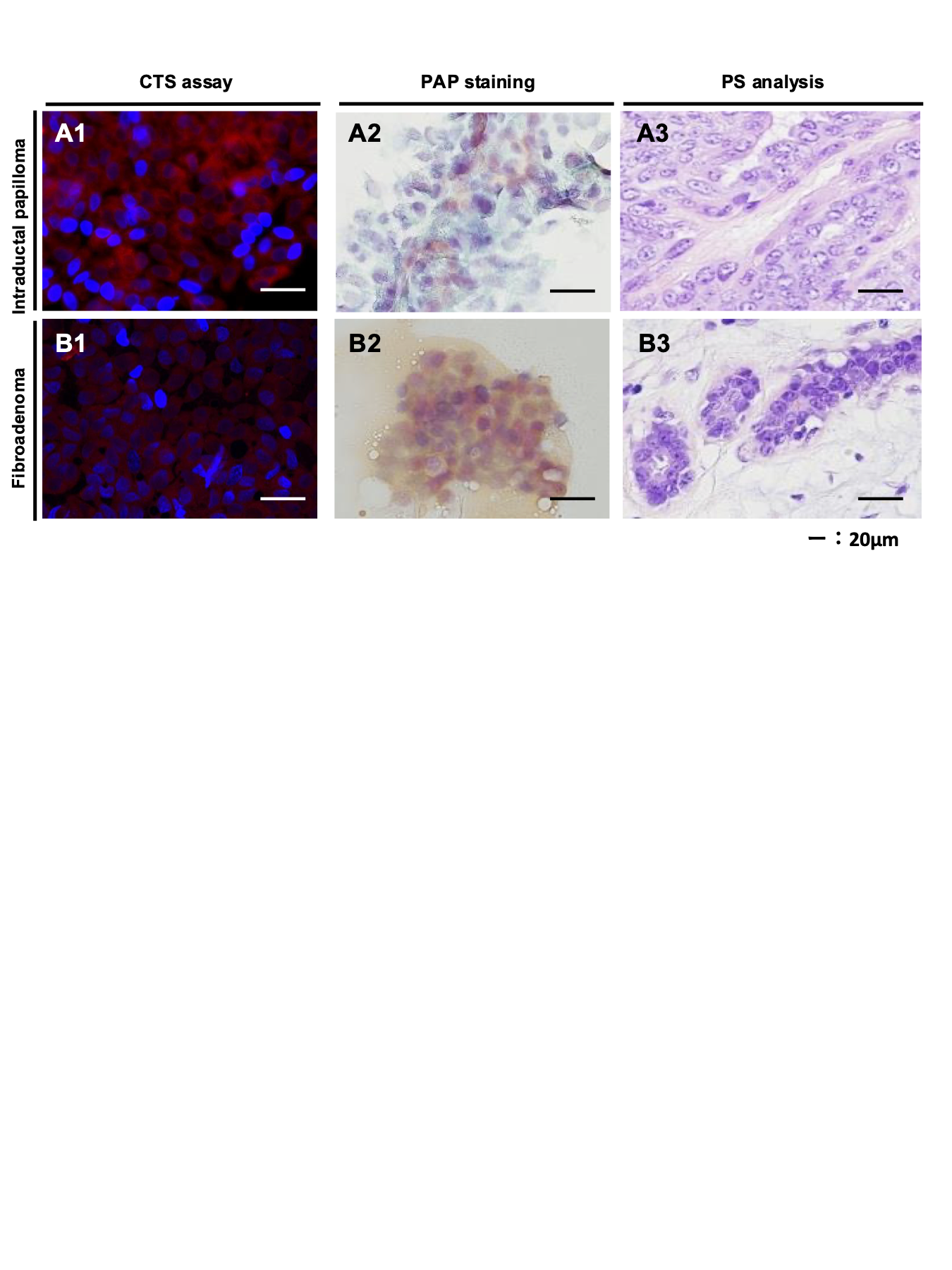

Supplement: Supplementary file 4 — Figure S4: Representative images of CTS assay false‐positive samples. CTS images were classified as positive by the CTS assay (A1–B1). On PAP stain analysis, A2 was classified as negative and B2 as positive. In contrast, the histopathological images (A3–E3) were diagnosed as intraductal papilloma (A1–A3) and fibroadenoma (B1–B3). CTS, click‐to‐sense; PAP, Papanicolaou; PS, permanent section. [file CAM4-15-e71525-s007.tiff]

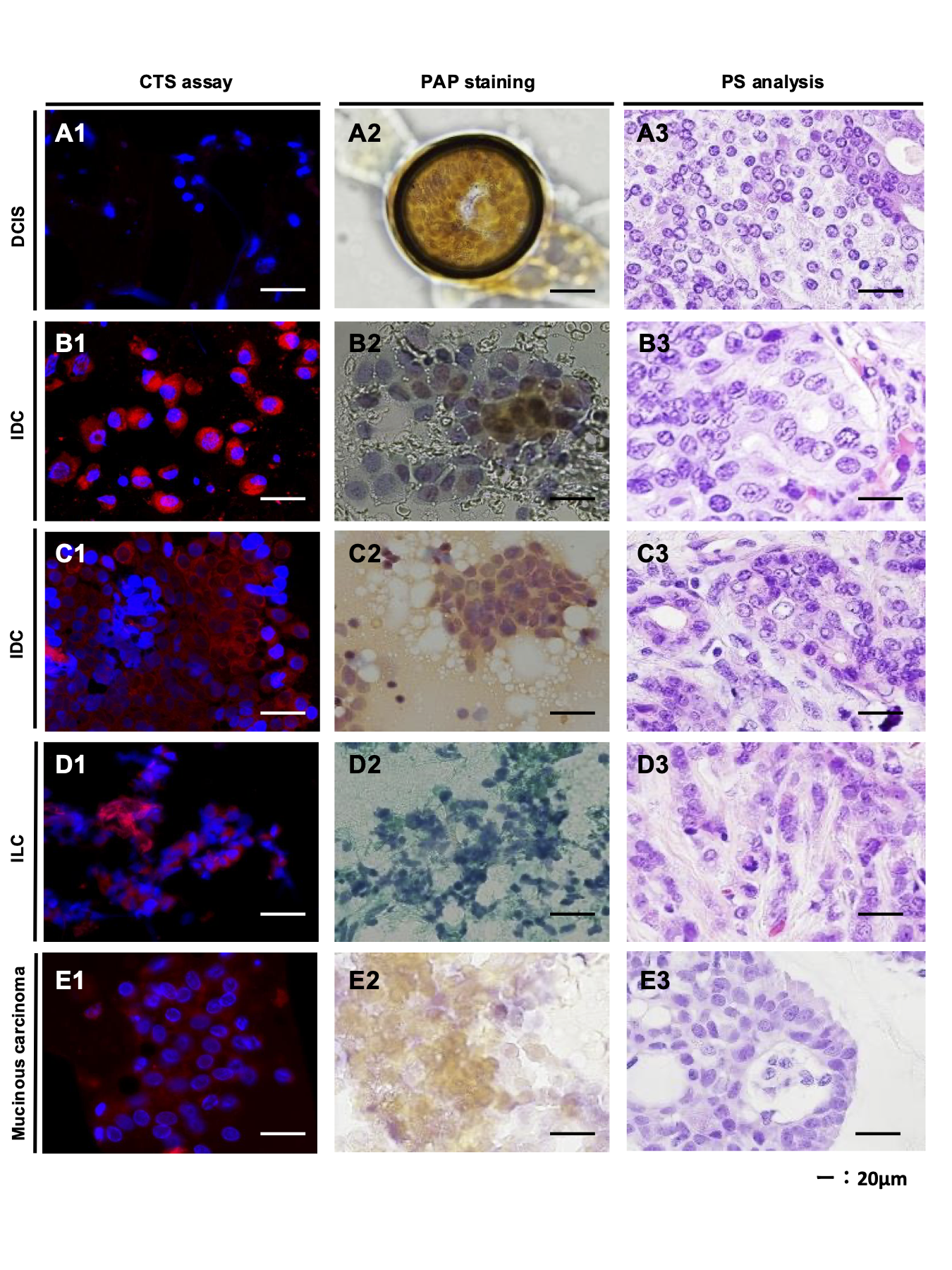

Supplement: Supplementary file 5 — Figure S5: Representative images of CTS assay false‐negative samples. CTS images were classified as negative by the CTS assay (A1–E1). On PAP stain analysis, A2–E2 was classified as positive. In contrast, histopathological images (A3–E3) were diagnosed as low‐grade DCIS (A1–A3), IDC (B1–B3, C1–C3), ILC (D1–D3), and mucinous carcinoma (E1–E3). CTS, click‐to‐sense; DCIS, ductal carcinoma in situ; IDC, invasive ductal carcinoma; ILC, invasive lobular carcinoma; PAP, Papanicolaou; PS, permanent section. [file CAM4-15-e71525-s008.tiff]

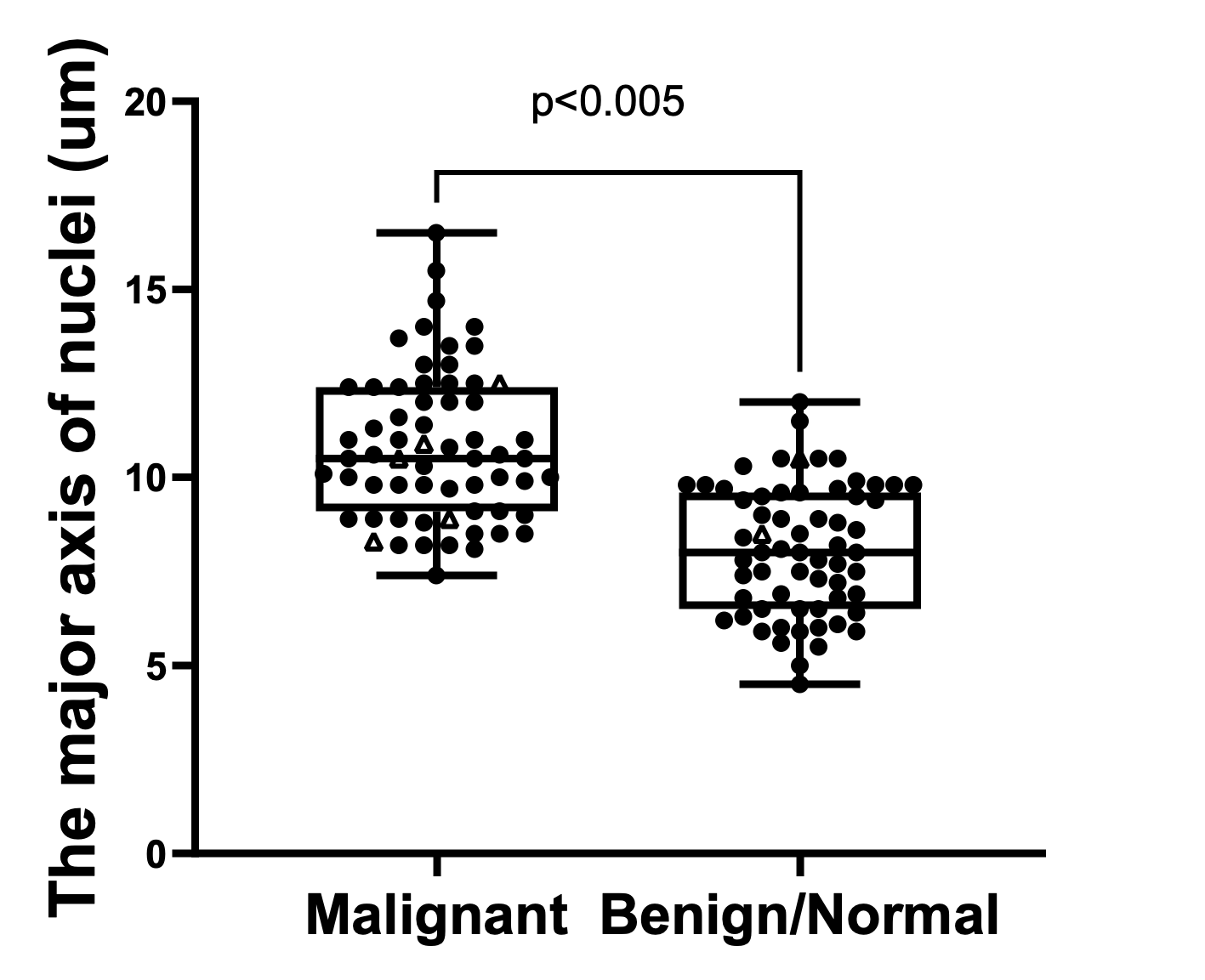

Supplement: Supplementary file 6 — Figure S6: The Major axis of nuclei in the 126 CTS images. The major axes of nuclei of representative cells were measured in the 126 CTS images. Δ, misclassification by the CTS assay. A statistically significant difference was observed between malignant and benign/normal breast tissues (p < 0.0001). [file CAM4-15-e71525-s009.tiff]
