# Supplementary material for: Rapid and Accurate Diagnosis of Breast Cancer by Fine‐Needle Aspiration Biopsy Using the “Click‐to‐Sense” Method
Source: Cancer Med. 2026 Feb 13;15(2):e71525. doi: 10.1002/cam4.71525 (PMC12903542; doi:10.1002/cam4.71525)
Supplement: Supplementary file 7 — Table S1: Results of the CTS assay and PAP staining analysis according to histological type. [file CAM4-15-e71525-s006.docx]

Supplementary Table S1. Results of the CTS assay and PAP staining analysis according to histological type

|  | N | |  | CTS assay | |  | PAP staining | | | |
| --- | --- | --- | --- | --- | --- | --- | --- | --- | --- | --- |
|  | n | % |  | Positive | Negative |  | Malignant/ Suspicious | Atypical | Benign | Insufficient/ Inadequate |
| **Patients** | 126 |  |  |  |  |  |  |  |  |  |
| **Malignant** | 63 |  |  | 58 | 5 |  | 61 | 0 | 1 | 1 |
| Ducal carcinoma in situ | 9 | 14.3 |  |  |  |  |  |  |  |  |
| High grade | 3 |  |  | 3 | 0 |  | 3 | 0 | 0 | 0 |
| Intermediate grade | 3 |  |  | 3 | 0 |  | 3 | 0 | 0 | 0 |
| Low grade | 3 |  |  | 2 | 1 |  | 3 | 0 | 0 | 0 |
| Invasive ductal carcinoma | 43 | 68.3 |  | 41 | 2 |  | 41 | 0 | 1 | 1 |
| Mucinous carcinoma | 5 | 7.9 |  | 4 | 1 |  | 4 | 0 | 0 | 0 |
| Invasive lobular carcinoma | 3 | 4.8 |  | 2 | 1 |  | 2 | 0 | 0 | 0 |
| Encapsulated papillary carcinoma with invasion | 1 | 1.6 |  | 1 | 0 |  | 1 | 0 | 0 | 0 |
| Apocrine carcinoma | 1 | 1.6 |  | 1 | 0 |  | 1 | 0 | 0 | 0 |
| Invasive micropapillary carcinoma | 1 | 1.6 |  | 1 | 0 |  | 1 | 0 | 0 | 0 |
| **Benign/Normal** | 63 |  |  | 2 | 61 |  | 6 | 2 | 51 | 4 |
| Fibroadenoma | 8 | 12.7 |  | 1 | 7 |  | 1 | 0 | 7 | 0 |
| Usual ductal hyperplasia | 8 | 12.7 |  | 0 | 8 |  | 2 | 1 | 3 | 2 |
| Intraductal papilloma | 6 | 9.5 |  | 1 | 5 |  | 1 | 1 | 3 | 1 |
| Mastopathy | 3 | 4.8 |  | 0 | 3 |  | 1 | 0 | 1 | 1 |
| Phyllodes tumor | 3 | 4.8 |  | 0 | 3 |  | 1 | 0 | 2 | 0 |
| Adenomyoepithelioma | 1 | 1.6 |  | 0 | 1 |  | 0 | 0 | 1 | 0 |
| Mucocele-like lesion | 1 | 1.6 |  | 0 | 1 |  | 0 | 0 | 1 | 0 |
| Normal | 33 | 52.4 |  | 0 | 33 |  | 0 | 0 | 33 | 0 |
| CTS, click-to-sense;  PAP, Papanicolaou | |  |  |  |  |  |  |  |  |  |
