# Supplementary material for: Rapid and Accurate Diagnosis of Breast Cancer by Fine‐Needle Aspiration Biopsy Using the “Click‐to‐Sense” Method
Source: Cancer Med. 2026 Feb 13;15(2):e71525. doi: 10.1002/cam4.71525 (PMC12903542; doi:10.1002/cam4.71525)
Supplement: Supplementary file 8 — Table S2: Number of samples diagnosed as positive by three diagnosticians working independently. [file CAM4-15-e71525-s003.docx]

Supplementary Table S2. Number of samples diagnosed as positive by three diagnosticians working independently

|  |  | PS analysis | | |
| --- | --- | --- | --- | --- |
|  |  | Malignant | Benign | Normal |
| CTS assay | Positive | 34 (3/3) | 0 (3/3) | 0 |
|  |  | 24 (2/3) | 2 (2/3) |  |
|  | Negative | 4 (1/3) | 9 (1/3) | 0 (1/3) |
|  |  | 1 (0/3) | 20 (0/3) | 32 (0/3) |
| CTS, click-to-sense; PS, permanent section | | |  |  |
