# Supplementary material for: Rapid and Accurate Diagnosis of Breast Cancer by Fine‐Needle Aspiration Biopsy Using the “Click‐to‐Sense” Method
Source: Cancer Med. 2026 Feb 13;15(2):e71525. doi: 10.1002/cam4.71525 (PMC12903542; doi:10.1002/cam4.71525)
Supplement: Supplementary file 9 — Table S3: Comparison of the CTS assay and PAP staining results. [file CAM4-15-e71525-s001.docx]

Supplementary Table S3. Comparison of the CTS assay and PAP staining results.

|  |  |  |  |  |  |  |
| --- | --- | --- | --- | --- | --- | --- |
|  |  | PAP stain | | | |  |
|  |  | Malignant/Suspicious | Atypical | Benign | Insufficient/  Inadequate | Concordance rate |
| CTS assay | Positive | 57 | 0 | 2 | 1 | 90.1% |
|  | Negative | 10 | 2 | 50 | 4 |  |
| CTS, click-to-sense;  PAP, Papanicolaou | | |  |  |  |  |
